# Super-enhancer profiling reveals ThPOK/ZBTB7B, a CD4^+^ cell lineage commitment factor, as a master regulator that restricts breast cancer cells to a luminal non-migratory phenotype

**DOI:** 10.1007/s00018-025-05913-4

**Published:** 2025-11-13

**Authors:** Camila D. Arcuschin, Kamin Kahrizi, Rosalyn W. Sayaman, Carolina DiBenedetto, Pedro J. Salaberry, Roxana Pirker, Yizhuo Shen, Ons Zakraoui, Cecilia Schwarzer, Alessandro Scapozza, Joseph A. Caruso, Paola Betancur, Julie D. Saba, Jean-Philippe Coppé, Mary-Helen Barcellos-Hoff, Dietmar Kappes, Laura van ‘t Veer, Ignacio E. Schor, Denise P. Muñoz

**Affiliations:** 1 Departamento de Fisiología, Biología Molecular y Celular, Facultad de Ciencias Exactas y Naturales, Universidad de Buenos Aires, Ciudad Universitaria, Buenos Aires, Argentina; 2 Instituto de Fisiología, Biología Molecular y Neurociencias, Universidad de Buenos Aires, CONICET, Ciudad Universitaria, Buenos Aires, Argentina; 3 Department of Pediatrics, Benioff Children’s Hospital at Oakland, University of California San Francisco, Oakland, CA 94609 USA; 4 Helen Diller Family Comprehensive Cancer Center, University of California San Francisco, San Francisco, CA 94115 USA; 5 Present Address: Lucira Health, Inc, Emeryville, CA 94608 USA; 6 Present Address: Harvard Medical School, Boston, MA 02115 USA; 7 Department of Biosciences, Heidelberg University, Im Neuenheimer Feld 234, 69120 Heidelberg, Germany; 8 School of Pharmaceutical Sciences, University of Geneva, 1 Rue Michel Servet, 1201 Geneva, Switzerland; 9 Fox Chase Cancer Center, Philadelphia, 19111 USA

**Keywords:** Transcriptional repressors, Gene regulatory networks, Extracellular matrix, Plasticity

## Abstract

Despite efforts to understand breast cancer biology, metastatic disease remains a clinical challenge. Identifying suppressors of breast cancer progression and mechanisms of transition to more invasive phenotypes could provide game changing therapeutic opportunities. Transcriptional dysregulation is central to all malignancies, highlighted by the extensive reprogramming of regulatory elements that underlie oncogenic programs. Among these, super-enhancers (SEs) stand out due to their enrichment in genes controlling cancer hallmarks. To reveal novel breast cancer dependencies, we integrated the analysis of the SE landscape with master regulator activity inference for a series of breast cancer cell lines. As a result, we identified **T**-**h**elper-inducing **Po**xviruses and **Z**inc-finger (**PO**Z)/**K**rüppel-like factor (ThPOK, *ZBTB7B*), a CD4^+^ cell lineage commitment factor, as a breast cancer master regulator that is recurrently associated with a SE. ThPOK expression is highest in luminal breast cancer but is significantly reduced in the basal subtype. Manipulation of ThPOK levels in cell lines shows that its repressive function restricts breast cancer cells to an epithelial phenotype by suppressing the expression of genes involved in the epithelial-mesenchymal transition (EMT), WNT/β-catenin target genes, and the pro-metastatic TGFβ pathway. Our study reveals ThPOK as a master transcription factor that restricts the acquisition of metastatic features in breast cancer cells.

**Supplementary Information:**

The online version contains supplementary material available at 10.1007/s00018-025-05913-4.

## Introduction

In recent years, breast cancer mortality has declined due to broader screening, early detection, better classification, and the discovery of new targeted therapies. However, metastatic breast cancer remains incurable, and many patients lack effective treatments [1, 2].

Breast cancer is commonly classified based on the expression of different receptors: estrogen and/or progesterone receptors (ER/PR +), amplification or overexpression of human epidermal growth factor 2 (HER2 +), or the absence of all three markers, which defines triple negative breast cancer (TNBC). Beyond this receptor-based classification, molecular intrinsic subtypes identified by the PAM50 gene expression signature further categorize breast cancers into luminal A (Lum A), luminal B (Lum B), HER2-enriched (HER2 +), basal-like, and normal-like subtypes [3, 4].

Both genetic and epigenetic alterations contribute to the heterogeneity of breast cancer, with aberrant epigenetic reprogramming playing a central role in tumor initiation and progression. Among these epigenetic features, super-enhancers (SEs), highly active regions of clustered enhancers, have emerged as critical regulatory elements in tumorigenesis, offering insights into cancer classification, subclonal evolution, and therapeutic response prediction [5, 6]. These regions are characterized by unusually high signals of histone modifications such as H3K27ac and H3K4me1, and chromatin regulatory proteins such as BRD4, the mediator complex, and cell-type specific transcription factors (TF) [7, 8]. SEs control the expression of genes essential for normal cells’ identity, and in cancer cells regulate genes involved in survival, oncogenesis and drug resistance [7–12]. This suggests that identifying SEs and their associated genes can reveal previously unappreciated cell dependencies and vulnerabilities, as well as inform about cancer biology, clinical diagnosis and therapeutic guidance [13]. In breast cancer cells (BCCs), SEs regulate genes involved in immune evasion [14] and therapeutic resistance [15, 16], and their perturbation by bromodomain and extraterminal (BET) protein inhibition restrains proliferation and promotes apoptosis [12, 17].

ThPOK (**T**-**h**elper-inducing **Po**xviruses and **Z**inc-finger (**PO**Z)/**K**rüppel-like factor), encoded by the *ZBTB7B* gene, is a transcription factor that belongs to the POK family of transcriptional repressors containing BTB/POZ domains. It was originally known for regulating extracellular matrix (ECM) genes in fibroblasts [18, 19] and CD4 + T lymphocytes lineage commitment [20], but its role in other contexts is less well known. While other members of the POK family have been implicated in carcinogenesis [21], there is no such oncogenic function described for ThPOK under non-manipulated circumstances. However, transgenic mice constitutively expressing ThPOK in T cells (T-cell specific *ThPOK* transgene, *ThPOK*^const^ mice) develop thymic lymphomas resembling human T-cell acute lymphoblastic leukemia (T-ALL) [22]. Only recently, ThPOK has been implicated in regulating lipid synthesis and lipid droplet secretion by the lactating murine mammary gland [23].

Here, we reveal a novel role for ThPOK as a master regulator in BCCs. The ThPOK/*ZBTB7B* gene is associated with a SE in cells derived from different breast tumor subtypes, a feature that distinguishes key transcriptional regulators in cancer. ThPOK expression levels are higher in breast tumors compared to normal adjacent tissues, with the highest levels observed in cells/tumors of the luminal subtype. Conversely, a major repressed status is observed in TNBC cell lines or tumors, likely driven by specific CpG methylated sites. Manipulation of ThPOK levels revealed a role for ThPOK in limiting BCCs plasticity to an epithelial non-migratory phenotype by restricting the expression of ECM genes, epithelial-mesenchymal transition (EMT) factors, and WNT and TGFβ pathways.

## Results

### Ubiquitous super-enhancers associate with key regulators of gene expression in breast cancer cell lines

**Fig. 1:**
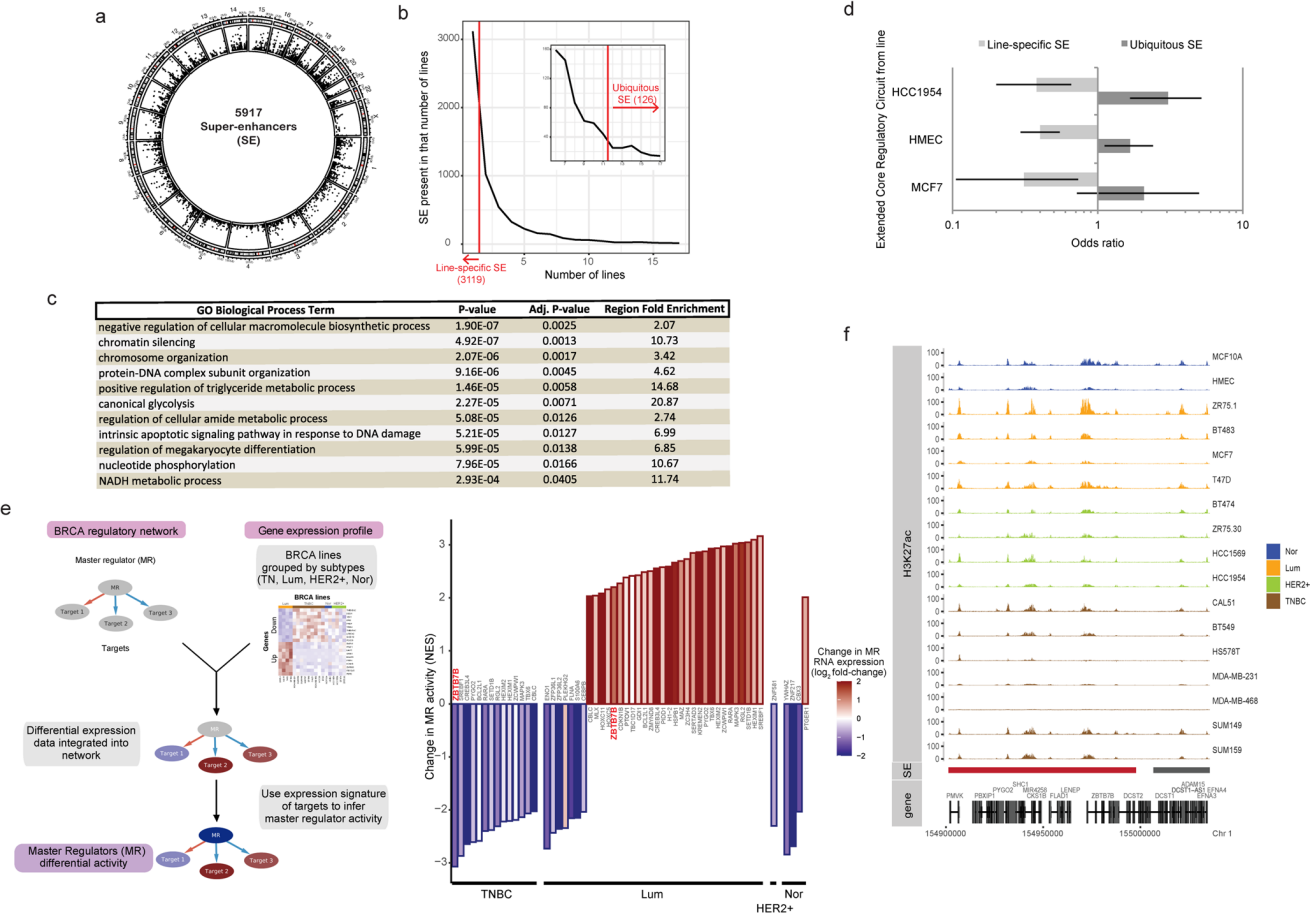
***Combining ubiquitous SE identification with subtype-specific master regulator activity*** ***analysis helps identification of candidate key*** ***regulators*** ***of breast cancer cell biology*.** **a)** Circo plot displaying the genome distribution of the whole set of 5917 SEs that resulted from the analysis of H3K27ac density from ChIP-seq data from 17 non-transformed mammary epithelial and BCC lines. Each of the boxes in the inner circle represents an instance of a SE in one cell line. **b)** Number of SEs as a function of the number of cell lines where each SE is present. Line-specific SEs, present in only one cell line, are shown left to the red line. The inset shows a magnification of the last part of the plot (from 6 to 17 cell lines). Ubiquitous SEs are present in more than two thirds of the cell lines (12 or more), right to the red line. **c)** Representative significant GO Biological Process terms enriched for genes associated with ubiquitous SEs vs all SEs, analyzed by GREAT. The full list of terms is shown in Table S4.** d)** Enrichment of ubiquitous SEs and depletion of line-specific SEs in the set of extended core regulatory circuitry (CRC) genes of three breast cell lines. The plot shows the odds-ratio with confidence intervals for a Fisher´s exact test (all *p*-values are < 0.05, except the one for MCF7 ubiquitous SEs). **e)** Analysis of differential activity for ubiquitous SE-associated master regulators (MR) of expression in breast cancer. *Left*: scheme of the VIPER analysis. Contrasts were made for each subtype (TNBC, Lum, HER2 + or normal) vs. the others. The heatmap for “Gene expression profile” represents the scaled expression across cell lines grouped by subtypes. Genes ordered by the gene expression signature resulting from one example contrast (Lum vs others), showing only the top 10 up- and down-regulated genes. *Right*: regulators with a normalized enrichment score (NES) from VIPER analysis of more than 2 in absolute value, shown for each contrast (lines of the indicated subtype vs all the other lines). The color scale of the bars shows the change in RNA expression of each MR for the indicated contrast. *ZBTB7B* (ThPOK) is indicated in red bold letters in the two contrasts where it appears (TNBC and Lum). **f)** Genomic context of *ZBTB7B* and its associated super-enhancer (red bar). The H3K27ac ChIP-seq signal is shown for all 17 breast cancer cell lines, grouped and colored by subtype

Since SEs control expression of oncogenes and regulators of cellular identity [5, 7, 24], we reasoned that characterizing SE profiles in both normal and cancer cells can reveal cancer-specific dependencies. To map the regulatory landscape of breast cancer, we used chromatin immunoprecipitation followed by sequencing (ChIP-seq) to determine the genome-wide distribution of H3K27ac in BCC lines derived from primary tumors. We combined our data with available H3K27ac ChIP-seq datasets from different BCC lines. After quality control of 80 input/H3K27ac ChIP-seq datasets, we analyzed 37 samples from 17 breast cancer cell lines (Table S1), identifying individual H3K27ac peaks and SE regions with MACS and ROSE2 algorithms, respectively [11, 25]. The consolidated set includes 5917 SEs (Fig. 1a) proximal (± 10Kbp) to 9911 genes. Our analysis confirmed that the 200 most variable SE regions (Table S2**)** cluster the cell lines according to their subtypes with few exceptions (Figs. S1a-b). Functional analysis of the complete SE set using the Genomic Regions Enrichment of Annotations Tool (GREAT) [26] showed SE-associated genes enriched in terms related to cell differentiation, mRNA metabolism, cell signaling and cell architecture (Fig. S1c and Table S3). In summary, using this strategy, we obtained a consolidated SE set that reflects known grouping of the cell lines and at the same time is associated with genes with functions relevant to carcinogenesis.

While many SEs within this set are only found in one cell line (cell line-specific SE, 3119), we identified a subset (126) of ubiquitous SEs that are common across at least two-thirds of the cell lines (Fig. 1b). GREAT analysis revealed that genes associated with ubiquitous SEs, unlike cell line-specific SEs, are significantly enriched in terms related to gene expression regulation, chromatin remodeling, differentiation, metabolism and apoptosis (Fig. 1c and Table S4). In addition, ubiquitous SEs are also specifically linked to BCC core regulatory circuitry genes (Fig. 1d, dark grey bars) defined in Saint-André et al. [24]. In conclusion, in contrast to cell line-specific SEs, we found that ubiquitous SEs are associated with key regulatory elements of breast cancer cell biology. Therefore, we focused on this subset for further analysis.

### ThPOK/ZBTB7B as a breast cancer master regulator repressed in TNBC

To prioritize key genes and cellular states, we used the ubiquitous SE set and integrated it with RNA-seq data from BCCs [27] (Table S5) to identify crucial transcriptional regulators with subtype-specific activity. We employed VIPER [28] to interrogate a breast cancer gene regulatory network built from The Cancer Genome Atlas (TCGA) transcriptomic data (ARACNe-BRCA) [29]. This revealed the activity patterns of 4438 breast cancer master regulators (MRs) by monitoring their downstream transcriptional targets expression (i.e. regulons) (Fig. 1e). By comparing each subtype (Lum, HER2 +, TNBC and normal (Nor)) to the rest, VIPER returned a normalized enrichment score (NES) for each MR in each subtype (Table S6). We focused on MRs with differential activity of absolute NES > 2 and genes associated with ubiquitous SEs, identifying 41 MR candidates (Fig. 1e, right panel). Many, but not all, showed differential activity that could be due in part to variations in their own expression (Fig. 1e, right panel, compare change in MR activity (bar height) vs change in RNA expression (bar color)). Notably, ThPOK (*ZBTB7B*), a CD4^+^ lineage commitment transcription factor, was significantly under-active in TNBC and over-active in luminal cell lines (Fig. 1e, right panel, red bold letters). This gene is linked to a large ubiquitous SE (Fig. 1f, red bar), though the H3K27ac signal near the transcriptional start site is lower in TNBC lines (Fig. 1f**, **Fig. S2a-b). Moreover, ThPOK fits the profile of a master regulator: it regulates gene expression, recruits chromatin repressors [30, 31], and influences cell lineage phenotypes [20, 32–34]. Given its oncogenic potential within the POK protein family, we investigated the specific role of ThPOK in breast cancer.

### ThPOK is upregulated in luminal breast cancer cells and tumors

**Fig. 2:**
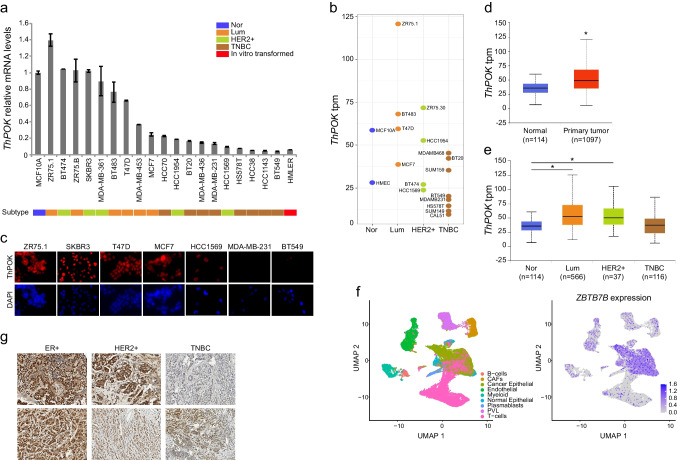
***ThPOK expression is highest in breast cancer cell lines and tumors of the luminal subtype.*** **a)** *ThPOK* expression in BCC lines measured by qRT-PCR. Bars and error bars represent the mean and the SD of ≥ 3 biological replicates. All values are referred to the average expression level in MCF10A cells. Breast cancer subtypes are colored as in Fig. 1: blue, Nor; orange, Lum; green, HER2 +; brown, TNBC; red, in vitro transformed cells. **b)** *ThPOK* mean expression (in TPM) extracted from RNA-seq datasets listed in Table S5. **c)** ThPOK protein expression assessed by immunofluorescence (red channel) in the indicated cell lines**.** Nuclei were counterstained with DAPI (blue channel). Images are shown at 400X magnification. **d-e)** *ThPOK* expression in primary tumors (TCGA dataset). Statistical analysis was performed by using Student’s *t*-Test with unequal variance. **d)** *ThPOK* expression is higher in primary tumors than in normal tissue, *: *p* < 10^–11^. **e)** *ThPOK* expression in breast cancer subtypes. Luminal tumors express the highest *ThPOK* levels compared to normal tissue *: *p* < 10^–12^, followed by HER2 + tumors, *: *p* < 10^–4^. *ThPOK* expression in TNBC tumors is not significantly different to the levels in normal tissues. In **d & e** in parenthesis: number of tumors/normal tissues included in each subtype. These plots were generated using the UALCAN portal (https://ualcan.path.uab.edu). **f)** ThPOK expression is higher in cancer epithelial cells within tumor samples. *ThPOK* levels in 38,241 cells from 11 ER + tumor samples assessed by scRNA-seq. Cell types were assigned following the identities from the original paper. **g)** Representative immunohistochemistry of ThPOK in breast carcinoma samples

Quantitative RT-PCR (qRT-PCR) analysis of *ThPOK* expression across 21 breast cancer cell lines revealed subtype-specific differences in mRNA levels: highest in luminal lines, lowest in TNBC lines, and intermediate yet more heterogeneous in HER2 + lines (Fig. 2a). These patterns were consistent with RNA-seq data from various cell lines (Fig. 2b) and suggested that, despite the ubiquitous presence of the *ZBTB7B/ThPOK*-associated SE (Fig. 1f), subtype-specific regulatory mechanisms modulate its expression, resulting in distinct mRNA abundance profiles. At the protein level, immunofluorescence confirmed a similar subtype-associated distribution of ThPOK protein (Fig. 2c). Therefore, both protein levels (Fig. 2c) and activity (Fig. 1e, right panel) agree with mRNA levels measured by qRT-PCR (Fig. 2a). Based on this correlation, we used qRT-PCR-based expression levels as a proxy for ThPOK activity in subsequent experiments.

To determine whether high *ThPOK* expression was driven by its ubiquitous SE, we inhibited BRD4, an essential SE component [7, 35, 36], using JQ1 or iBET-151. Acute BRD4 inhibition (6 h) reduced *ThPOK* expression in three cell lines (T47D, MCF7 and BT474) with moderate-to-high endogenous levels (Fig. S2c, left), without affecting the housekeeping gene *ACTB* (Fig. S2c, right). In contrast, *ThPOK* expression driven by a stably integrated lentiviral construct in cell lines with low endogenous ThPOK (MDA-MB-231 and HCC1143) remained unchanged upon treatment (Fig. S2d), indicating that BRD4 inhibition specifically impacted the endogenous *ZBTB7B*/*ThPOK* SE-driven transcription.

Analysis of RNA-seq data from patient samples in TCGA, accessed through the UALCAN portal [37, 38], showed higher *ThPOK* expression in breast tumors compared to adjacent normal tissue (Fig. 2d). Subtype comparisons revealed elevated ThPOK mRNA levels in luminal hormone receptor + (HR +) and HER2 + tumors relative to TNBC (Fig. 2e). Single cell RNA-seq data from ER + tumors [39] further confirmed high *ThPOK* expression specifically in cancer epithelial cells (Fig. 2f). To validate our expression findings at the protein level in patient tissues, we performed immunohistochemistry (IHC) for ThPOK across breast tumor samples. Consistent with mRNA profiling and immunofluorescence in cell lines, IHC revealed markedly higher ThPOK staining intensity in ER + tumors compared to HER2 + and TNBC subtypes (Fig. 2g). Corresponding IgG control staining and hematoxylin and eosin for the tumor samples shown in Fig. 2g are provided in Fig. S3 (panels a and b, respectively). This subtype-associated protein expression pattern reinforces the notion that ThPOK is most abundantly expressed in luminal tumors, supporting its potential relevance in hormone receptor-positive breast cancer.

Detailed analysis of TCGA data, incorporating PAM50 classification [4] and hormone receptor status determined by immunohistochemistry (Fig. S3c-d), showed the highest *ThPOK* mRNA levels in PAM50 LumA and HER2 + subtypes (Fig. S3c). In contrast, more aggressive PAM50 LumB tumors had lower *ThPOK* levels (Fig. S3c). Among receptor subtypes, ER^+^ tumors showed the highest *ThPOK* levels, with HER2 + tumors displaying the highest *ThPOK* expression in the ER^−^ group (Fig. S3d). Epigenetic analysis revealed increased CpG methylation at several sites within the *ZBTB7B* 5’ UTR in TNBC tumors, suggesting transcriptional repression mediated by CpG methylation (Figs. S3.e-g). Supporting the significance of ThPOK’s role in breast cancer, data from the Human Protein Atlas highlighted that breast cancer exhibited the highest ThPOK protein levels (Fig. S4a) and ranked second for mRNA expression across cancer types (Fig. S4b). Clinical relevance of ThPOK activity was further supported from the analysis of an independent dataset, the METABRIC cohort [40]. Consistent to what we observed in TCGA expression data (Fig. S3d), this analysis indicated that ThPOK’s activity is indeed elevated in ER + tumors (Fig. S5a). Notably, low ThPOK activity was associated with worse prognosis (higher Nottingham Prognostic Index) (Fig. S5b) and higher probability of positive lymph nodes (Fig. S5d), two associations that were specific for ER + tumors (Fig. S5c, e). In addition, relapse-free survival was significantly lower for tumors with low ThPOK activity (Fig. S5f), a trend that was also specific to ER + samples (Fig. S5g). Additionally, we observed that deletions of the *ZBTB7B* gene correlated with shorter time to metastasis (Fig. S5h-i, Metastatic Breast Cancer Project data).

### ThPOK silencing de-represses a pro-migratory gene expression program

**Fig. 3:**
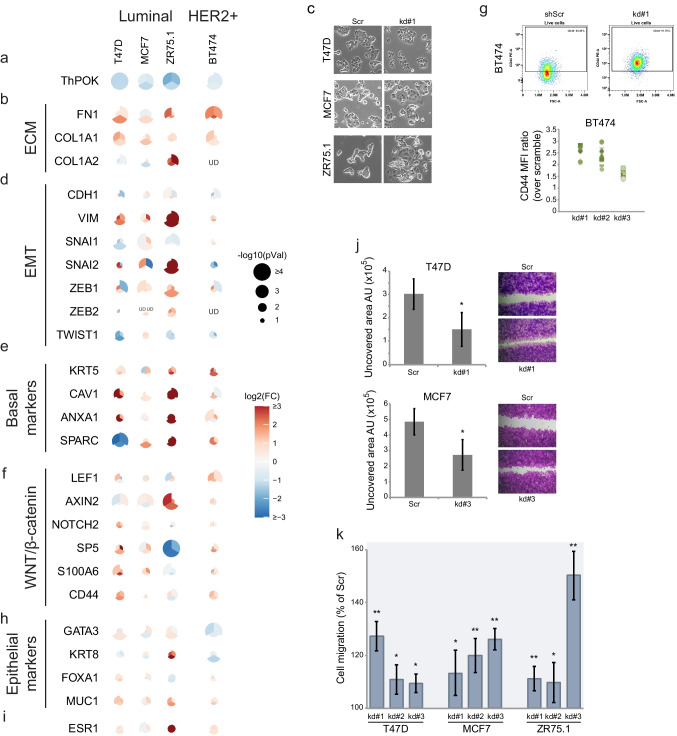
***ThPOK knockdown dysregulates expression of ECM, EMT and WNT/β-catenin genes and increases migration of luminal BCCs.*** **a, b, d-f, h-i)** Gene expression levels measured by qRT-PCR. Since three different shRNA against *ThPOK* were used, the circle for each gene is divided in thirds, one for each shRNA. The color of each third indicates the log_2_(fold-change) and the size the -log_10_(p-value), for a *t*-test relative to the control shRNA (shScr). Each gene was assessed in ≥ 3 biological replicates. Differential expression is shown in color scale as a log2 of the fold change, and *p*-values by the size of the circle as –log10. **c)** Bright field images show morphological changes associated with ThPOK-kd in Luminal and HER2 + cells (kd#1 was used as representative for all cell lines). Scale: each side of the square corresponds to 400 µm. **g)** Flow cytometry analysis showing CD44 expression in HER2 + scramble (Scr) and ThPOK-kd BT474 cells. **j-k)** Migration assays. **j)** Scratch assay showing increased migratory features of ThPOK-kd cells compared with control (scr) cells. Bar and error bars represent the mean and the SD of ≥ 3 biological replicates. *: *p* < 10^–6^. **k)** Boyden chamber assay. Bar and error bars represent the mean and the SD of ≥ 3 biological replicates, *: *p* < 0.05, **: *p* < 10^–4^. All *p-*values in this figure were calculated based on a two-tailed Student’s *t*-Test

Although ThPOK is mainly known as a transcriptional repressor, depending on the context it can also act as an activator [30, 33, 41, 42]. To investigate ThPOK’s specific functions and downstream targets in BCCs, we performed shRNA-mediated knockdown (kd) to reduce its mRNA and protein levels (Fig. 3a and S6a) in luminal (T47D, MCF7 and ZR75.1) and HER2 + (BT474) cell lines exhibiting hightointermediate *ThPOK* mRNA expression (Fig. 2a). We first examined the expression of known ThPOK target genes previously identified in fibroblasts and T cells, as some of these may also be relevant in breast cancer. Knocking down ThPOK levels led to increased ECM gene expression (*FN1*, *COL1A1*, *COL1A2*) (Fig. 3b), and caused EMT-like morphological changes in the assessed cell lines (Fig. 3c, S6b). Since ThPOK has features of a master regulator, we assessed the impact of its silencing on other gene sets representing different cancer hallmarks. ThPOK-kd led to an increase in the expression of EMT factors (*VIM*, *SNAI2*, *ZEB1*, *ZEB2*), basal markers and WNT/β-catenin target genes implicated in breast cancer cell proliferation (Fig. 3d-f) [43, 44]. The protein membrane and stemness marker CD44 was also upregulated as shown by qRT-PCR (Fig. 3f) and flow cytometry analysis (Fig. 3g and S6c-d). Consistent with this, using TCGA breast tumor’s RNA-seq data, we found that low levels of ThPOK correlate with a higher RNA-based stemness score [45] (Fig. S6e). We did not observe a consistent change in expression of epithelial markers (Fig. 3h) but the *ESR1* gene, coding for the ER, was upregulated both in luminal and HER2 + cell lines (Fig. 3i) in response to ThPOK-kd. In agreement with the observed alteration of their transcriptional programs, luminal cells with reduced levels of ThPOK exhibited an enhanced migratory phenotype (Fig. 3j-k and S6f).

### ThPOK expression induces a “luminal-like” gene signature in HER2 + and TNBC cell lines

**Fig. 4:**
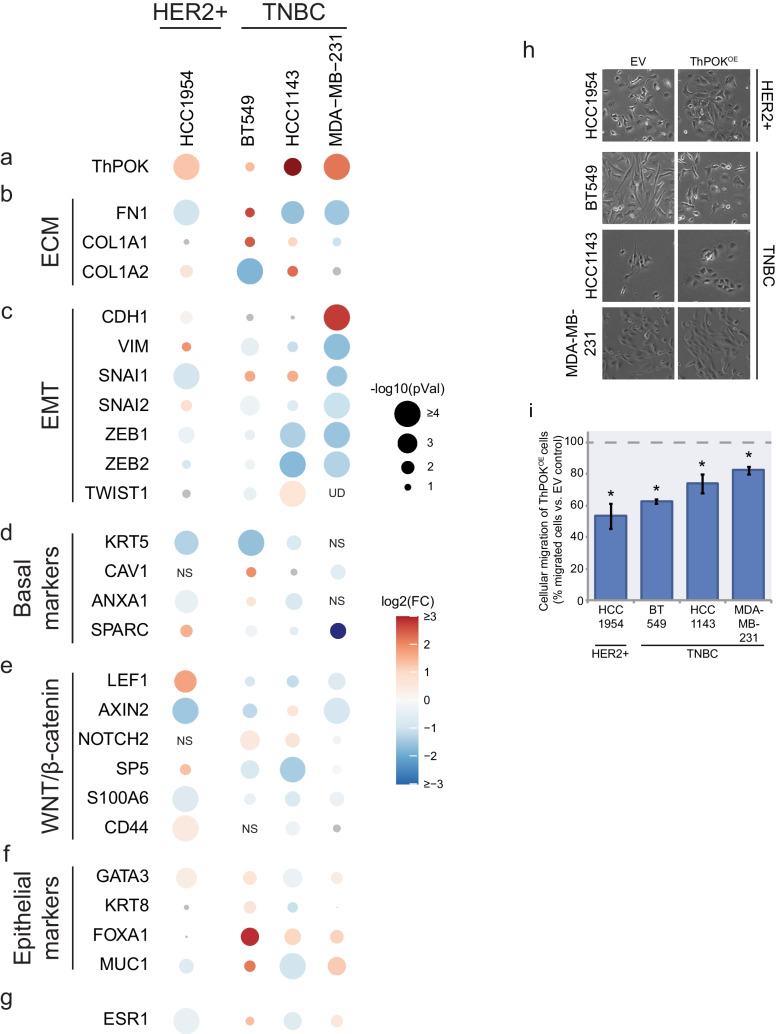
***ThPOK overexpression represses ECM, EMT and WNT/β-catenin genes and decreases migration in HER2 + and TNBC BCCs. *a-g)** Gene expression levels measured by qRT-PCR. The log_2_(fold-change) in expression of each gene is shown in color scale and p-values by the size of the circle as –log_10_, relative to the empty vector control cells (EV). Each gene was assessed in ≥ 3 biological replicates. **h)** Bright field images show morphological changes associated with ThPOK overexpression in HER2 + and TNBC cells. Scale: each side of the square corresponds to 400 µm. **i)** Boyden chamber migration assay. Cellular migration was assessed by measuring the absorbance of crystal violet released by migrated cells in each condition. Data are expressed as a percentage relative to EV control cells. The dashed line indicates the 100%, corresponding to the migration level of EV cells. Bars and error bars represent the mean and SD of ≥ 3 biological replicates, *: *p* < 10^–6^. *p-*value was calculated based on a two-tailed Student’s *t*-Test

Based on our previous findings, we hypothesized that overexpressing ThPOK in HER2 + and TNBC cells with low endogenous ThPOK levels would elicit opposite changes in gene expression and cellular phenotypes. We transduced HCC1954 (HER2 +), and HCC1143, BT549 and MDA-MB-231 (TNBCs) with a lentivirus encoding ThPOK variant #5, the most abundant variant expressed in BCCs (Fig. S7a-b). ThPOK overexpression (ThPOK^OE^) was confirmed by qRT-PCR and Western blot (Fig. 4a and Fig. S8a). As predicted, ThPOK overexpression decreased ECM gene expression (Fig. 4b) and increased epithelial markers including *CDH1* (Figs. 4c, f), while reducing the expression of genes associated with migration and invasiveness, such as EMT markers (*VIM*, *SNAI1*, *SNAI2*, *ZEB1*, *ZEB2* and *TWIST*) (Fig. 4c), basal markers (Fig. 4d), WNT/β-catenin target genes and the stemness marker *CD44* (Fig. 4e), as well as *ESR1* (Fig. 4g). These changes are accompanied by a mesenchymal to epithelial transition-like phenotype and reduced migratory features (Figs. 4h-i and S8b).

Taken together, these data indicate that ThPOK functions as a transcriptional repressor of genes that are critical for breast cancer phenotypes associated with invasiveness and aggressiveness.

### ThPOK inhibits the TGFβ pathway but independently regulates ECM genes

**Fig. 5:**
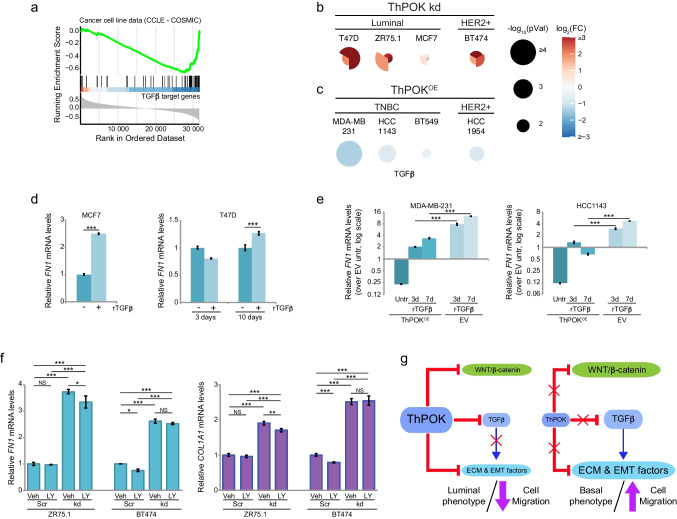
***ThPOK represses TGFβ expression in breast cancer cell lines.*** **a)** GSEA analysis shows an inverse correlation between ThPOK expression levels and TGFβ target genes. Genes were ordered according to their expression correlation with *ZBTB7B* using transcriptomic data from CCCL (breast cancer cell lines). TGFβ target genes are enriched at the end of the ordered set (negative correlations). **b-c)** TGFβ (*TGFB1*) mRNA levels measured by qRT-PCR. **b)** TGFβ levels in ThPOK-kd luminal and HER2 + BCCs. *TGFB1* expression in each independent shRNA-containing cells is depicted as a triangle and the fold change is relative to that of the control shRNA (shScr). Each gene was assessed in ≥ 3 biological replicates. Differential expression is shown in color scale as a log_2_ of the fold change, and p-values by the size of the circle as –log_10_. **c)** TGFβ levels in HER2 + and TNBC cells overexpressing ThPOK (ThPOK^OE^). *TGFB1* expression is shown as the fold change relative to that of the empty vector control cells (EV). Each gene was assessed in ≥ 3 biological replicates. Differential expression is shown in color scale as a log_2_ of the fold change, and p-values by the size of the circle as –log_10_. **d)** *FN1* relative mRNA levels measured by qRT-PCR in MCF7 cells after 3 days treatment with recombinant human TGFβ (rTGFβ), and T47D cells after 3 or 10 days treatment with rTGFβ. **e)** *FN1* relative mRNA levels measured by qRT-PCR in MDA-MB-231 and HCC1143 cells with ThPOK^OE^ or EV, untreated (Untr) or treated with rTGFβ for 3 or 7 days. **f)** *FN1* and *COL1A1* relative mRNA levels measured by qRT-PCR in Scr and ThPOK-kd (kd) ZR75.1 and BT474 cells, treated with DMSO (Veh) or TGBRI inhibitor (LY). For **d**-**f**, all expression values are depicted relative to the mean of the corresponding controls. Bars and error bars represent the mean and the SD of ≥ 3 biological replicates. NS: non-significant; *: *p* < 0.05; **: *p* < 0.005; ***: *p* < 0.0005. All *p-*values in this figure were calculated based on a two-tailed Student’s *t*-Test. **g)** Proposed model showing the direct and indirect regulation of extracellular matrix, EMT and TGFβ/SMAD target genes’ expression by ThPOK. The size of the balloons represents the relative contribution (not in scale) of each factor to the cellular phenotypes

Since the expression of *FN1* and *COL1A1* genes is positively regulated by TGFβ signaling [42], we hypothesized that the repressive action of ThPOK over these ECM genes could be through downregulation of the TGFβ pathway, a central regulator of ECM remodeling and fibrosis. Indeed, when we analyzed the levels of TGFβ/SMAD target genes in breast cancer cell lines from the Cancer Cell Line Encyclopedia (CCLE), we saw an inverse correlation between the expression of *ThPOK* and of TGFβ target genes (Fig. 5a). By measuring TGFβ (*TGFB1*) mRNA levels in ThPOK-kd (Fig. 5b) and ThPOK^OE^ cells (Fig. 5c) by qRT-PCR, we confirmed that ThPOK is a repressor of TGFβ mRNA expression in BCCs. TGFβ is produced as a latent complex and is activated extracellularly by many factors [46]. To test whether ThPOK-kd cells can activate the produced TGFβ, control cells were exposed to conditioned media collected from ThPOK-kd cultures in the presence or absence of a TGFβ receptor I (TβRI) inhibitor (LY2157299), and expression of *FN1* and *COL1A1* genes was assessed. Conditioned media from ThPOK-kd cells significantly increased ECM genes’ mRNA levels in luminal/HER2 + cells, and this effect was mostly mitigated by TβRI inhibition (Fig. S9a), indicating that ThPOK-kd cells produce and activate TFGβ, which may have autocrine as well as paracrine effects on ECM genes’ expressions.

We hypothesized that if ThPOK represses ECM genes by repressing the expression of TGFβ, ThPOK action should be bypassed by exogenous TGFβ treatment. However, while treatment of luminal cancer cell lines with recombinant TGFβ (rTGFβ) increased *FN1* and *COL1A1* expression, the magnitude of the effect was smaller, and a longer treatment was needed in cells with high endogenous ThPOK levels (T47D), compared to cells with lower levels (MCF7) (Figs. 5d and S9b). Similarly, rTGFβ treatment only partially counteracted the effects of ectopic ThPOK overexpression in TNBC cell lines (MDA-MB-231 and HCC1143) for the assessed ECM genes (Figs. 5e and S9c). These results suggest that in both scenarios, the repression imposed by high levels of endogenous and/or ectopic ThPOK in the expression of ECM genes it can only be partially counteracted by the inducing effects of TGFβ signaling, suggesting a direct regulatory role of ThPOK on these genes, besides its action through the regulation of *TGB1* expression. To further confirm this, we inhibited TβRI activity in ThPOK-kd luminal and HER2 + cells, so that any autocrine signaling would be blunted, and evaluated *FN1* and *COL1A1* expression. Although TβRI inhibition slightly reduced the expression of TGFβ target genes, it did not fully revert their levels to those of the original scramble control cells (Fig. 5f, compare Veh vs kd + LY). This implies that while TGFβ produced by ThPOK-kd cells may autocrinally contribute to some extent to the increase in ECM gene expression when ThPOK levels are reduced, the primary driver of their dysregulation is the absence of ThPOK’s repressive functions.

In summary, our studies highlight ThPOK as a key transcriptional regulator that limits breast cancer cells plasticity by repressing TGFβ, ECM genes and mesenchymal factors, thereby promoting an epithelial, non-migratory phenotype (Fig. 5g).

## Discussion

Epigenetic dysregulation is a hallmark of cancer, and the identification of superenhancers (SEs) has aided in the discovery of unexpected dependencies and novel drug targets [8, 36]. In this study we employed a multi-omic approach that integrates SE profiling and transcriptomic analysis of 17 normal mammary and breast cancer cell lines with a breast cancer-specific gene regulatory network, revealing ThPOK/*ZBTB7B* as a subtype-specific master regulator in breast cancer (Fig. 1).

Our study presents ThPOK, a zinc-finger transcriptional repressor known for its role in T cell lineage commitment [20], as a potential regulator of luminal identity. Clinically, lower ThPOK expression and copy number loss are associated with worse prognosis in ER + tumors, including shorter relapse-free survival and earlier metastasis (Fig. S5). In luminal contexts, ThPOK appears to help maintain epithelial features, while its lower expression in more aggressive subtypes coincides with activation of transcriptional programs associated with tissue remodeling and immune evasion. Finally, silencing of ThPOK expression in high-expressing cells, such as luminal cell lines, increases their migratory capacity (Fig. 3), while its overexpression in low-expressing cells, such as TNBC and certain HER2 + cell lines, ameliorates their migration properties (Fig. 4), showing a causal effect of ThPOK on tumoral phenotype.

A key step in our analysis was the prioritization of a small subset of SEs, referred to as ubiquitous SEs, which consistently showed SE features in the different BCC lines assessed and were enriched in the proximity of key cell identity genes (Fig. 1c-d). We believe that selecting for commonalities between cell lines helped us to avoid epigenetic abnormalities that may arise in specific cell lines and do not necessarily represent properties of that tumor type. At the same time, even though the *ZBTB7B* gene was associated with a ubiquitous SE, it showed clear subtype-specific expression. This may indicate that SEs impart complex regulatory architecture rather than solely controlling gene expression, potentially granting chromatin accessibility at the ThPOK locus that allows fine-tuning to adapt to the changing demands of the lactating mammary gland. Further functional analysis of the individual regulatory elements within the *ZBTB7B* SE are required to fully understand the impact of the SE chromatin conformation in the context of cell-type specific expression for this gene.

Our discovery of ThPOK as a master regulator is further supported by its identification as a methylation modulator gene in breast invasive carcinoma [47], based on an integrated analysis of DNA-methylation and RNA-seq data from approximately 500 tumor samples. In this dataset, ThPOK emerged as a hub node within a gene module enriched for WNT/β-catenin and TGFβ signaling targets [47]. Its inverse correlation with the stemness index and CD44 expression (Fig. 3f-g and S6c-e) further supports a role for ThPOK in restricting cellular plasticity and promoting a differentiated state. While ThPOK has traditionally been studied in the context of hematopoiesis and T-cell fate [48, 49], its emerging role in epithelial tumor biology highlights the importance of lineage-specific transcription factors in shaping tumor identity and evolution. Our work expands the repertoire of known ThPOK functions, positioning it as a regulatory "brake" that stabilizes epithelial identity in breast cancer and limits plasticity-driven tumor progression.

ThPOK may operate through multiple layers of regulation, including suppression of TGF-β signaling and direct repression of many targets, which contribute to migration and immune evasion [50–52]. This includes upregulation of key ECM components such as fibrillar collagens and FN1, many of which are canonical targets of TGF-β signaling and are commonly associated with aggressive, invasive breast cancer behavior [44, 53].

Mechanistically, ThPOK physically interacts with histone deacetylases (HDACs) and is enriched at promoters of non-transcribed genes, supporting that it primarily functions as a transcriptional repressor [30, 41]. This role, consistent with the idea of a regulatory brake, is supported by the results of our knockdown (Fig. 3) and overexpression experiments (Fig. 4), which indicate ThPOK-mediated repression of genes involved in ECM composition, as well as transcription factors implicated in the EMT and WNT/β-catenin signaling pathways. These effects coincided with cellular morphological and behavioral changes indicative of altered migratory capacity. A direct regulatory role of ThPOK over these genes remains to be fully resolved, and chromatin immunoprecipitation (ChIP-seq) or CUT&RUN approaches are necessary to determine whether ThPOK binds directly to promoters or enhancers of key targets such as *FN1*, *TGFB1*, and *COL1A1*. In addition, we identified a common set of genes regulated by ThPOK and Polycomb repressor complex 2 (PRC2) in transformed mammary epithelial cells [54]. Since both, dysfunction of PRC2 [54] and ThPOK knockdown (this study), lead to a quasi-mesenchymal state characterized by de-repression of many EMT and basal genes and retention of some epithelial features, it is possible that a cooperation between ThPOK and proteins of the Polycomb family are needed to exert some of its repressive functions.

Importantly, our identification of ThPOK as a repressive regulator adds to the growing recognition of the role of transcriptional repressors in tumor suppression. While much of cancer biology has focused on activating oncogenic programs, our data highlight the importance of maintaining repressive networks, the disruption of which can facilitate malignant transformation.

The ECM influences cancer cell behavior through chemical and mechanical signals, with changes in collagen composition linked to tumor invasion and aggressiveness [55]. Recent studies have shown that collagen deposition and organization regulate dormancy in disseminated breast cancer cells and immune exclusion in TNBC [56, 57]. Given ThPOK’s repression of ECM genes, the regulation of ThPOK expression could profoundly impact the ECM and consequently alter cancer cell behavior. This is also consistent with the fact that ThPOK regulates *TGFB1* mRNA and TGFβ signaling pathways which play a critical role in tumor formation, progression and metastasis [44]. Notably, *FN1* and *COL1A1*, all regulated by TGFβ [50, 51], exhibit coordinated expression in breast cancer lymph node metastases [51]. These proteins form networks that promote a spindle-like phenotype, significantly enhancing tumor cell migration, and are associated with poor outcomes [51]. While ThPOK-mediated repression of ECM genes could occur indirectly through autocrine TGFβ signaling, our findings (Fig. 5), together with previous studies [18, 19], also suggest a direct role for ThPOK in the transcriptional inhibition of these genes, indicating that this mechanism is at least partially independent of TGFβ regulation.

While ThPOK expression is enriched in luminal breast cancers and downregulated in more aggressive subtypes, restoring its function —or mimicking its repressive effects— could hold therapeutic value. Tumors in hybrid EMT states, which are often therapy-resistant and immunosuppressive [58, 59], may be particularly responsive to ThPOK-mimetic strategies aimed at restoring epithelial features. Although direct replacement poses challenges, targeting the downstream effects of ThPOK loss offers a potential path for limiting metastasis and improving therapeutic response. This therapeutic value might not be limited to breast cancer, since similar roles for ThPOK have been proposed in other cancers: in gastric cancer CTSL^High^/ZBTB7B^Low^ expression marked poor outcomes [60], while in hepatocellular carcinoma, ZBTB7B loss promoted dedifferentiation and tumor initiation via c-Jun-mediated reprogramming [61]. Together, these data suggest a conserved function for ThPOK in stabilizing epithelial identity and repressing plasticity across tumor types.

A subset of ductal carcinoma in situ (DCIS) cases progress to invasive ductal carcinoma (IDC) [62, 63], posing clinical challenges in the identification of patients requiring a more aggressive treatment. Investigating ThPOK repression as a mechanism facilitating the acquisition of more aggressive phenotypes could shed light on those progressive cases that transition to invasive disease. The application of spatial transcriptomics and single-cell epigenomic profiling would help capture the dynamic and heterogeneous regulation of ThPOK, particularly in processes like the ductal carcinoma in situ (DCIS) to invasive ductal carcinoma (IDC) transition, where spatial context and cellular heterogeneity are critical.

Finally, since our conclusions are based on cell culture models and correlative data on patients, an in vivo validation of ThPOK mechanism is required. Mouse models engineered for ThPOK modulation in mammary epithelium will be essential to define its role in tumor initiation, progression, immune modulation, and metastasis.

In summary, our study suggests a tumor-suppressive role for ThPOK in breast cancer, where it represses EMT and ECM gene programs, helps maintain luminal identity, and limits cellular plasticity. Its downregulation may promote tumor progression through epigenetic and microenvironmental mechanisms. These findings highlight the broader relevance of transcriptional repressors in constraining aggressive phenotypic transitions and raise the possibility of using ThPOK as a biomarker of progression and plasticity.

## Materials and methods

### Cell lines, culture conditions, constructs and lentivirus production and transduction

HCC1954, BT20, BT483, HCC1569, and BT474 breast cancer cell lines were bought from ATCC. MDA-MB-361 and MDA-MB-453 were bought from UC Berkeley Cell Culture laboratory. ZR75.1, MDA-MB-436, and ZR75.B were kindly given by Dr. Desprez. MCF10A, MCF7, T47D, MDA-MB-231, and SKBR3 were kindly given by Dr. Kohwi-Shigematsu. HMLER cells were kindly provided by Dr. Weinberg. All cell lines tested negative for Mycoplasm, were grown at 5%CO_2_ with all media was supplemented with 10% FBS and 1X Penicillin/Streptomycin. BT20, BT474, HS578T, MCF7, MDA-MB-231, MDA-MB-453, MDA-MB-361, MDA-MB-436, and SKBR3 were grown in DMEM; BT483, BT549, HCC1143, HCC38, HCC70, HCC1569, HCC1954 and ZR75.1, MDA-MB-436, ZR75.B, and T47D in RPMI. MCF10A and HMLER cells were grown as described [64, 65].

### ThPOK construct

*ThPOK* mRNA variant #5 (NCBI Reference Sequence: NM_001256455.2) was amplified by PCR from cDNA and cloned into pENTR1A-DS vector (Invitrogen) using SalI and NotI sites, then sequenced for accuracy. The pENTR1A-ThPOK vector was recombined into pLenti-CMV-DEST-hygro (W117-1) (provided by Dr. Campeau [66]) using Clonase II (Thermofisher, cat# 11,791).

### Lentivirus production and transduction

shRNA-expressing lentiviral vectors for ThPOK knockdown were purchased from Open Biosystems/Dharmacon (RHS4533-EG51043). Lentiviruses containing shRNA or ThPOK constructs were produced as described in Rodier et al. [67]. Transductions were done overnight with 4 µg/ml of Polybrene™. 48 h later cells were selected with 2 µg/ml puromycin or 400 µg/ml hygromycin.

### Chromatin immunoprecipitation and next generation sequencing (NGS)

Chromatin preparation and immunoprecipitation were performed as described in [68] using a H3K27ac antibody (Abcam cat# Ab4729). ChIP-seq libraries were prepared using Illumina protocols, quantified with Kapa Biosystems kits, pooled and sequenced on a HiSeq4000 as 50-base single reads.

### NGS data analysis

RNA-seq data were quality controlled with FastQC. Reads were aligned and transcript counts were obtained using kallisto with 100 bootstraps [69]. Master regulator activity inference from RNA-seq data was performed with VIPER [28]. ChIP-Seq data were quality controlled with FastQC, mapped using Bowtie 2.0, and H3K27ac peaks and SE were obtained using MACS2 and ROSE2 algorithms [7, 8, 25]. For more detail refer to supplementary methods.

### Analysis of single cell RNA-seq data from tumors

Processed scRNA-seq data of 11 IHC-inferred ER + breast cancer tumors from [39] was downloaded from the Gene Expression Omnibus (GEO) under accession number GSE176078 and was analyzed in R with the Seurat suite [70] using standard pipelines. Gene expression data of each tumor was normalized using scTransform and consequently integrated with the canonical correlation analysis (CCA) method using default parameters. Cell type identification was taken from the original paper. To visualize the single-cell data in a reduced-dimensional space, we performed Principal Component Analysis (PCA) and Uniform Manifold Approximation and Projection (UMAP) with 20 dimensions and k.param = 20 for FindNeighbors and n.neighbors = 20 for RunUMAP. Plots were generated with the functions DimPlot and FeaturePlot. *ZBTB7B*/ThPOK levels were expressed in normalized counts by scTransform.

### Immunofluorescence and immunohistochemistry

ThPOK immunofluorescence was performed as previously described [71], using an antibody from Novus Biologicals cat# NBP1-88077. Secondary antibody was anti-rabbit IgG ReadyProbes™ Alexa Fluor 594. Nuclei were counterstained with DAPI. Images were taken using the same exposure time using a Keyence fluorescence microscope BZ-X800 at 400X magnification.

For ThPOK immunohistochemistry, formalin-fixed, paraffin-embedded tissue sections from the Cooperative Human Tissue Network (CHTN) Western Division, Nashville, TN, were baked at 60 °C for 1 h. Slides were deparaffinized sequentially in Xylene (2 × 7 min), followed by rehydration through a graded ethanol series (100% ethanol, 2 × 7 min; 95% ethanol, 10 min; 70% ethanol, 10 min), and rinsed in distilled water for 5 min. Slides were then baked again at 60 °C for 15 min. Antigen retrieval was performed in a Tris-based antigen unmasking solution (Vector Laboratories, H-3301) for 25 min, followed by cooling to room temperature for 30 min. All subsequent washes were carried out using 1% Tween-20 in Tris-buffered saline (TBST). Slides were washed in TBST for 5 min, then endogenous peroxidase activity was quenched using BLOXALL blocking solution (Vector Laboratories, SP-6000) for 10 min, followed by another TBST wash. Non-specific binding was blocked by incubation in 5% bovine serum albumin (BSA-IgG free) in PBS for 20 min. Following a TBST wash, sections were incubated overnight at 4 °C with anti-ThPOK antibody (Cell Signaling Technology 13205S) diluted 1:100 in 1% BSA in PBS or normal rabbit IgG (0.4 µg/ml – sc-2067). After a TBST wash, slides were incubated for 30 min with a biotinylated anti-rabbit IgG secondary antibody (Vector Laboratories, BA-1000) diluted 1:200 in 1% BSA in PBS. Following an additional TBST wash, signal amplification was achieved using the VECTASTAIN® Elite ABC-HRP Kit (Vector Laboratories, PK-6100) for 30 min. Detection was performed using 3,3'-diaminobenzidine (DAB; Vector Laboratories, SK-4103) for 7 min, followed by a 5 min rinse in tap water. Slides were counterstained with hematoxylin (Vector Laboratories, H-3404) for 30 s, rinsed, and mounted with VectaMount AQ aqueous mounting medium (Vector Laboratories, H-5501). Hematoxylin and eosin staining was performed by following the protocol described in Fischer et al. [72]. Slides were imaged using Aperio AT2 at 20x.

### Western blot

Cells were lysed and proteins separated by SDS-PAGE. Primary antibodies: ThPOK (Novus Biologicals cat# NBP1-88077); GAPDH (Cell Signaling cat# 97166); β-Actin (Abcam cat# ab6276). Secondary antibodies: anti-mouse and anti-rabbit HRP-conjugated (BioRad cat# 1705047EDU and 1662408EDU).

### RNA extraction, cDNA synthesis and qRT-PCR

RNA was purified with TRIzol followed by DNAse RNase-free digestion (Qiagen). cDNA synthesis was performed using the High-Capacity cDNA Reverse Transcription kit (ABI). qRT-PCR primers sequences will be provided upon request. qRT-PCR was performed using SYBR-Green Power master mix (Thermofisher), analyzed with the QuantStudio5 software, and relative mRNA levels were calculated using the 2^^(−∆∆Ct)^ method using *GAPDH* and *HPRT1* as controls. We used a *t*-test for two-samples with equal variance (with a two-tailed distribution) to test the significance of gene expression differences.

### Flow cytometry

For CD44 staining, cells were collected, washed in FACS buffer (2% FBS in PBS), and kept at 4ºC for the rest of the protocol. Cells were stained for 1 h with an anti-CD44-PE antibody (Cell Signaling, cat# 88151) at a 1:300 dilution in FACS buffer. After washing, cells were stained for 20 min with LIVE/DEAD Fixable Violet Dead (Life Technologies) at a 1:7500 dilution in PBS. After washing, CD44 + events were quantified using a Northern Light cytometer (Cytek). Data were analyzed on SpectroFlo software (Cytek).

### Scratch and Boyden chamber assays

For the scratch assay, once cells reached confluency three parallel scratches were made per well. After 48 h, cells were fixed with 4% (V/V) paraformaldehyde, stained with 0.1% Crystal Violet for 20 min, rinsed and air-dried. The scratches were imaged using a Nikon Eclipse TS100 light microscope under 4 × objective. Multiple and overlapping images were taken along each scratch. ImageJ was used to measure the area unoccupied by cells, which was reported under the measurement parameter “Area”. We created Macro Plug-in with a code to analyze the images in batches. Data from individual images were grouped together per scratch. Images with highly irregular shapes and of the head and the tail region of the scratches were excluded. The assay results of each scratch were calculated by taking the mean of those grouped images, and then plotting based on different treatments. Data were obtained from at least 3 biological replicates. Boyden chamber assay was performed as described in [71] with no Matrigel and 10% FBS-media as chemoattractant. Cells on the outside bottom of the wells were fixed with 4% paraformaldehyde, stained with 0.1% crystal violet, extracted with 10% acetic acid and quantified at 590 nm. Experiments were done three times in triplicates.

### Treatment with TGFβ and inhibition of TGBRI

Cells were treated with 10 ng/ml of rTGFβ (Life Technologies, cat#: PHG9204) for the indicated times. To assess the activity of TGFβ produced by ThPOK-kd cells, the cells were grown in RPMI/DMEM with 10% of Serum Replacement Medium (KO SRM Gibco cat# 10828–028) for 72 h before the supernatant was collected, centrifuged and filtered to constitute the conditioned media (CM). shScr cells were grown in the presence of conditioned media with or without the TGBRI inhibitor LY2157299, at a 2 nM concentration for 24 h. To assess TGFβ’s autocrine function, cells were grown in RPMI/DMEM with 10% SRM for 24 h with or without the TGBRI inhibitor LY2157299 at a 2 nM concentration.

### GSEA and stemness score analyses

CCLE (https://sites.broadinstitute.org/ccle/datasets) dataset was used to calculate Spearman correlations between *ZBTB7B* and the rest of the genes, and the correlation-ranked genes were analyzed for enrichment of TGFβ target genes [73] using GSEA [74] within the clusterProfiler R package [75]. Samples were divided in two groups based on the median value of *ZBTB7B* RNA expression. t-test with Welch’s correction was performed.

## Supplementary Information

Below is the link to the electronic supplementary material.

Supplementary file1 (PDF 1735 KB)

Supplementary file2 (PDF 1040 KB)

Supplementary file3 (PDF 10093 KB)

Supplementary file4 (PDF 657 KB)

Supplementary file5 (PDF 15484 KB)

Supplementary file6 (PDF 7620 KB)

Supplementary file7 (PDF 663 KB)

Supplementary file8 (PDF 14154 KB)

Supplementary file9 (PDF 427 KB)

Supplementary file10 (XLSX 18 KB)

Supplementary file11 (XLSX 1619 KB)

Supplementary file12 (XLSX 333 KB)

Supplementary file13 (XLSX 66 KB)

Supplementary file14 (XLSX 10 KB)

Supplementary file15 (XLSX 887 KB)

Supplementary file16 (DOCX 709 KB)

## Data Availability

Data publicly available analyzed in this study is listed with their references in Supplementary Tables 1 & 5. Datasets generated in the current study have been deposited in the GEO repository, https://www.ncbi.nlm.nih.gov/geo/query/acc.cgi?acc=GSE207245.
