# Supplementary figures and images for: Super-enhancer profiling reveals ThPOK/ZBTB7B, a CD4+ cell lineage commitment factor, as a master regulator that restricts breast cancer cells to a luminal non-migratory phenotype
Source: Cell Mol Life Sci. 2025 Nov 13;82(1):397. doi: 10.1007/s00018-025-05913-4 (PMC12615902; doi:10.1007/s00018-025-05913-4)

## Supplementary Figure 1

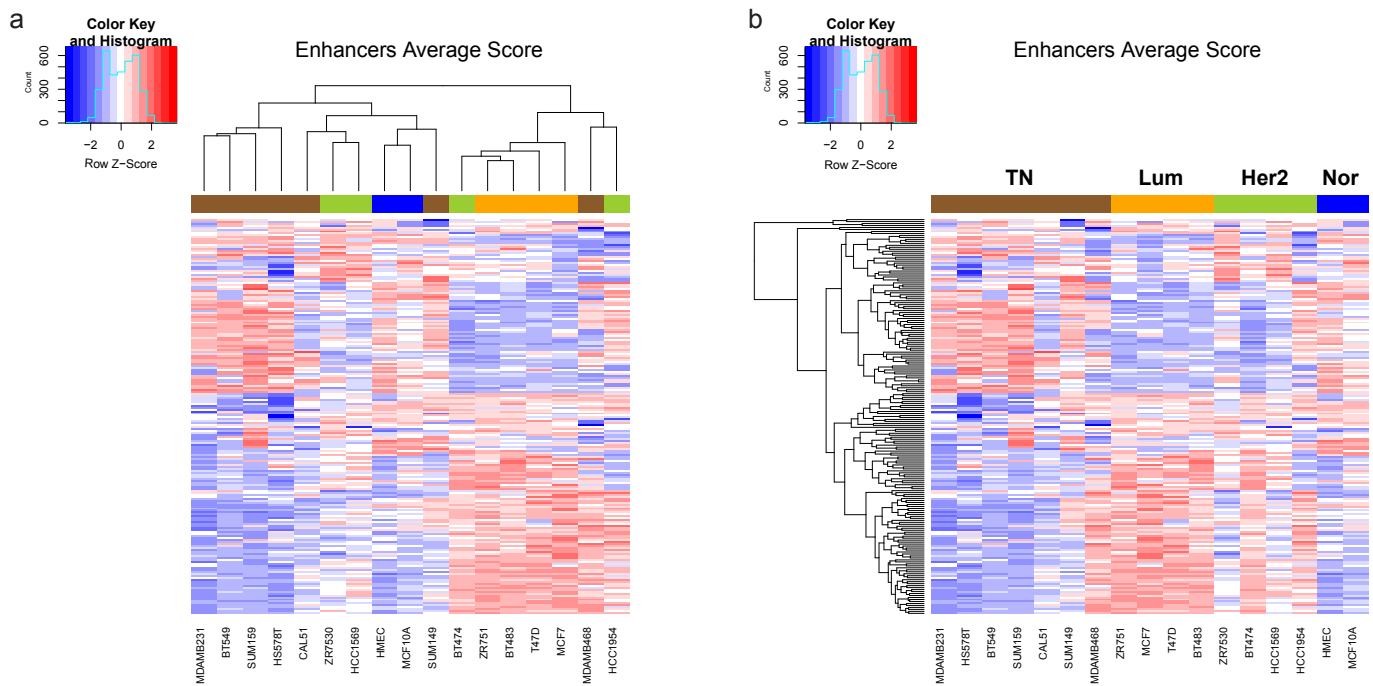

## C

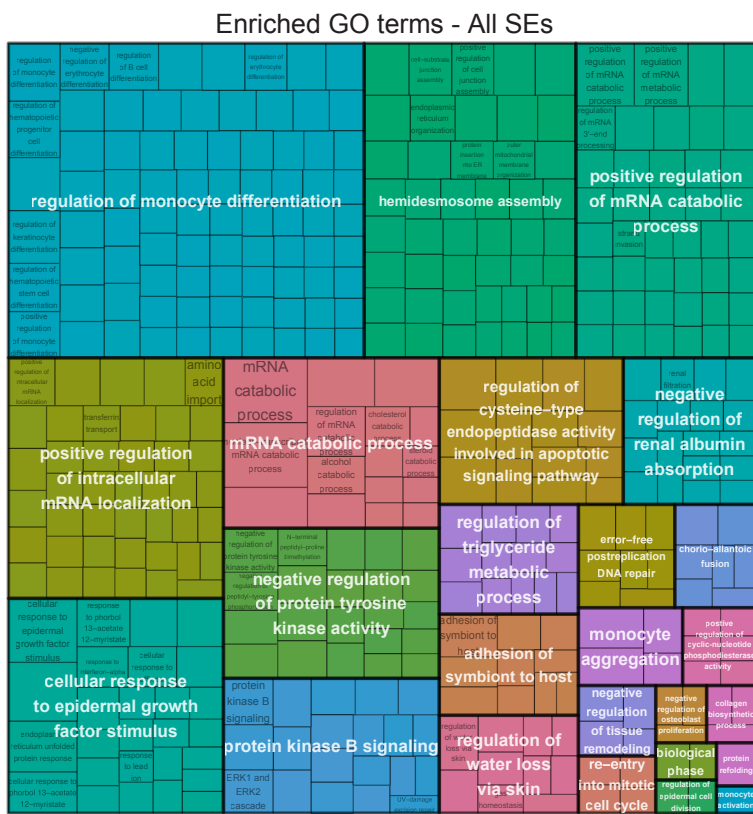

Supplement: Supplementary file 1 — Supplementary file1 (PDF 1735 KB) [file 18_2025_5913_MOESM1_ESM.pdf]

# Supplementary Figure 3

a

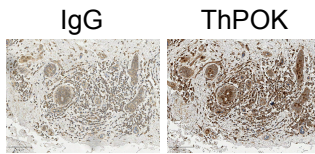

b

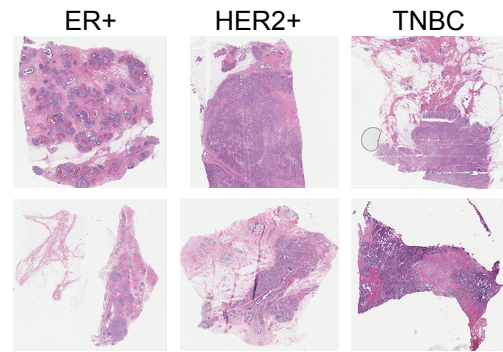

c

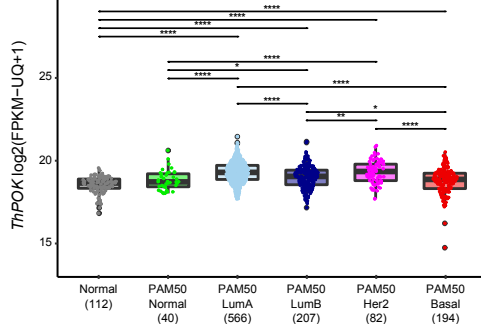

d

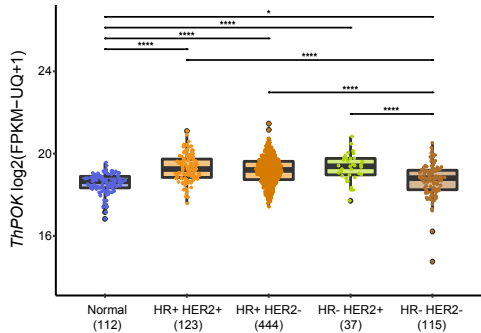

e

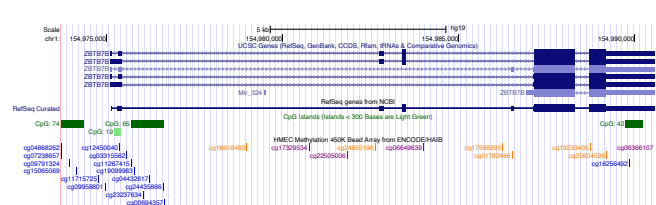

f

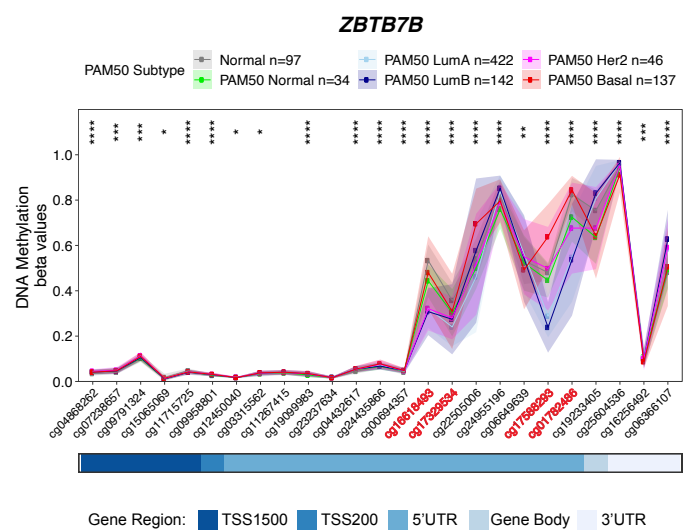

g

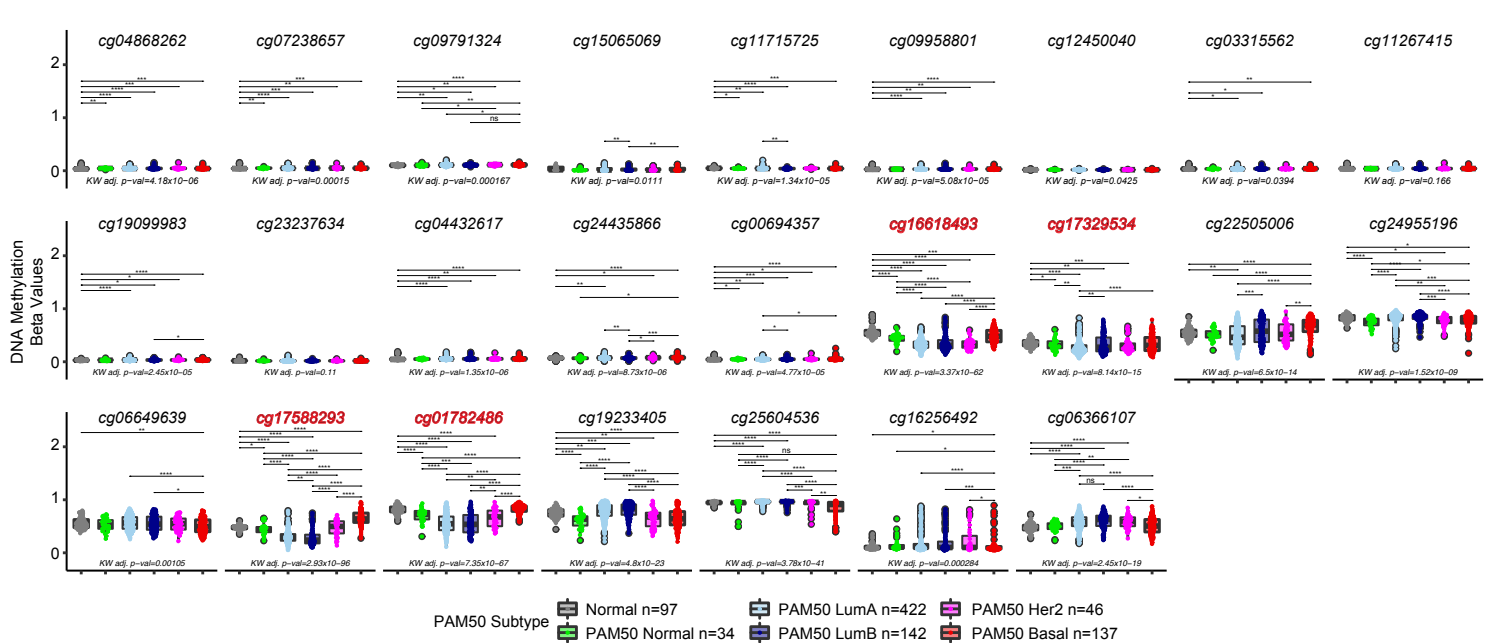

Supplement: Supplementary file 3 — Supplementary file3 (PDF 10093 KB) [file 18_2025_5913_MOESM3_ESM.pdf]

Supplementary Figure 4

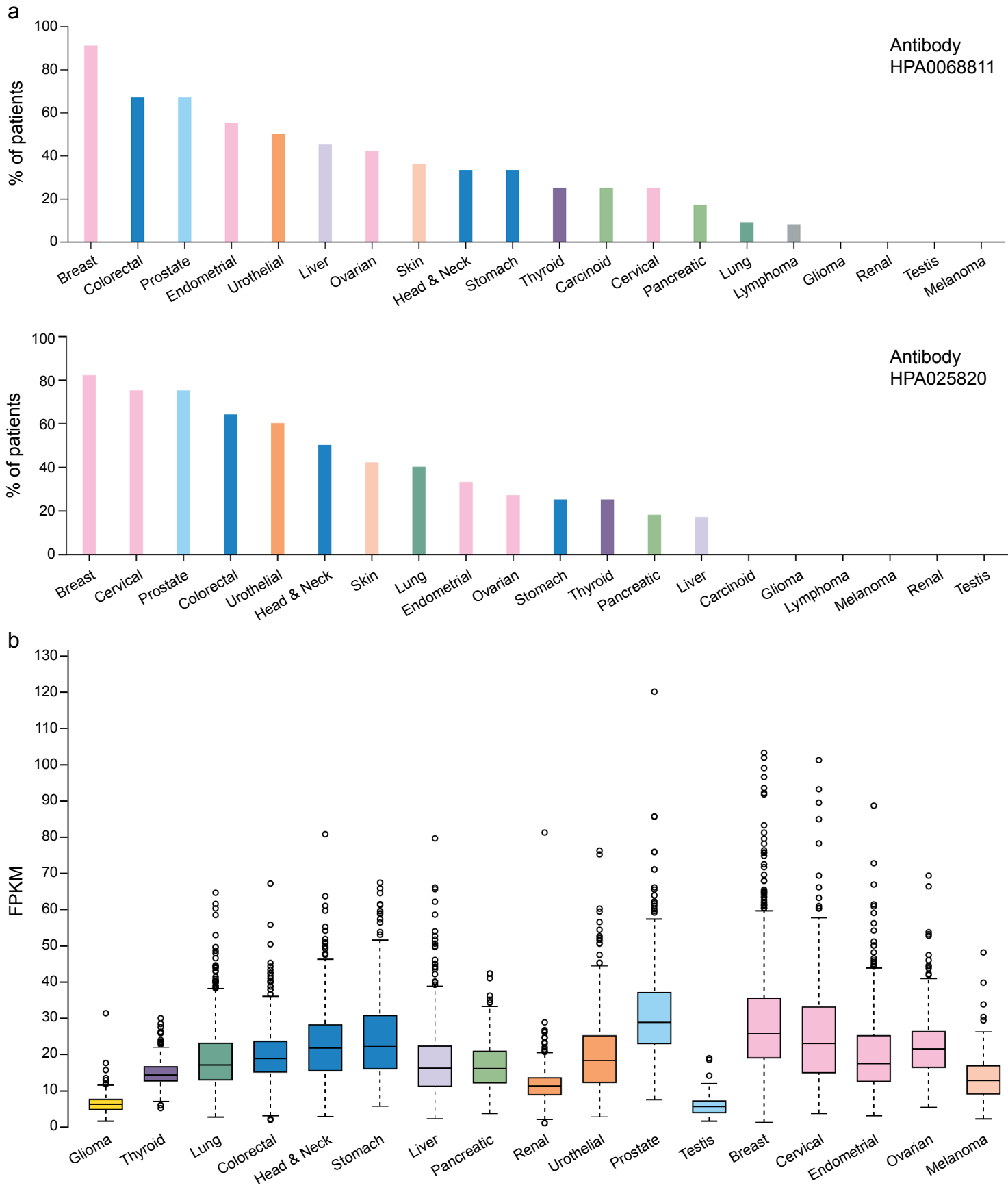

Supplement: Supplementary file 4 — Supplementary file4 (PDF 657 KB) [file 18_2025_5913_MOESM4_ESM.pdf]

Supplementary Figure 5

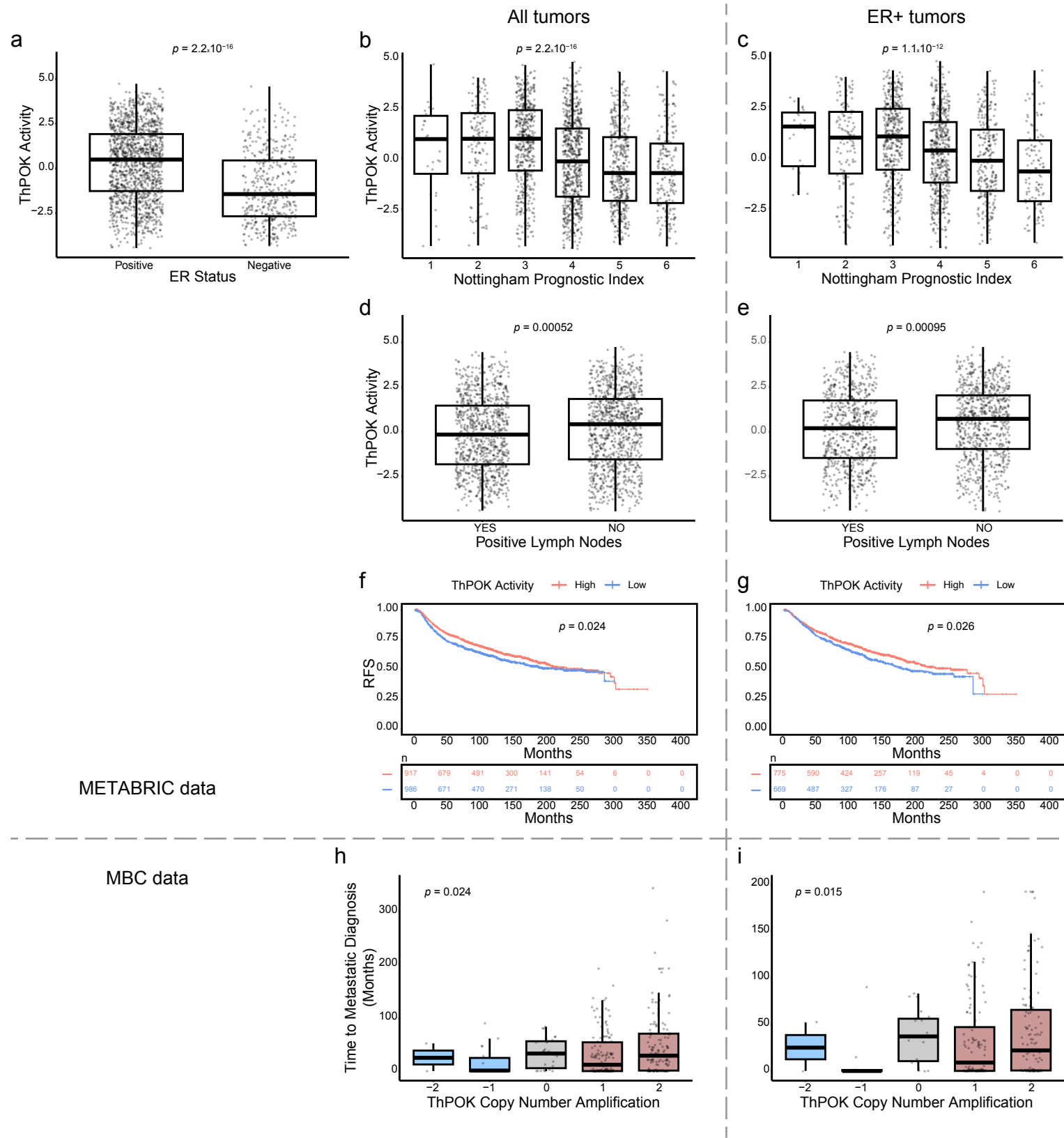

Supplement: Supplementary file 5 — Supplementary file5 (PDF 15484 KB) [file 18_2025_5913_MOESM5_ESM.pdf]

Supplementary Figure 6

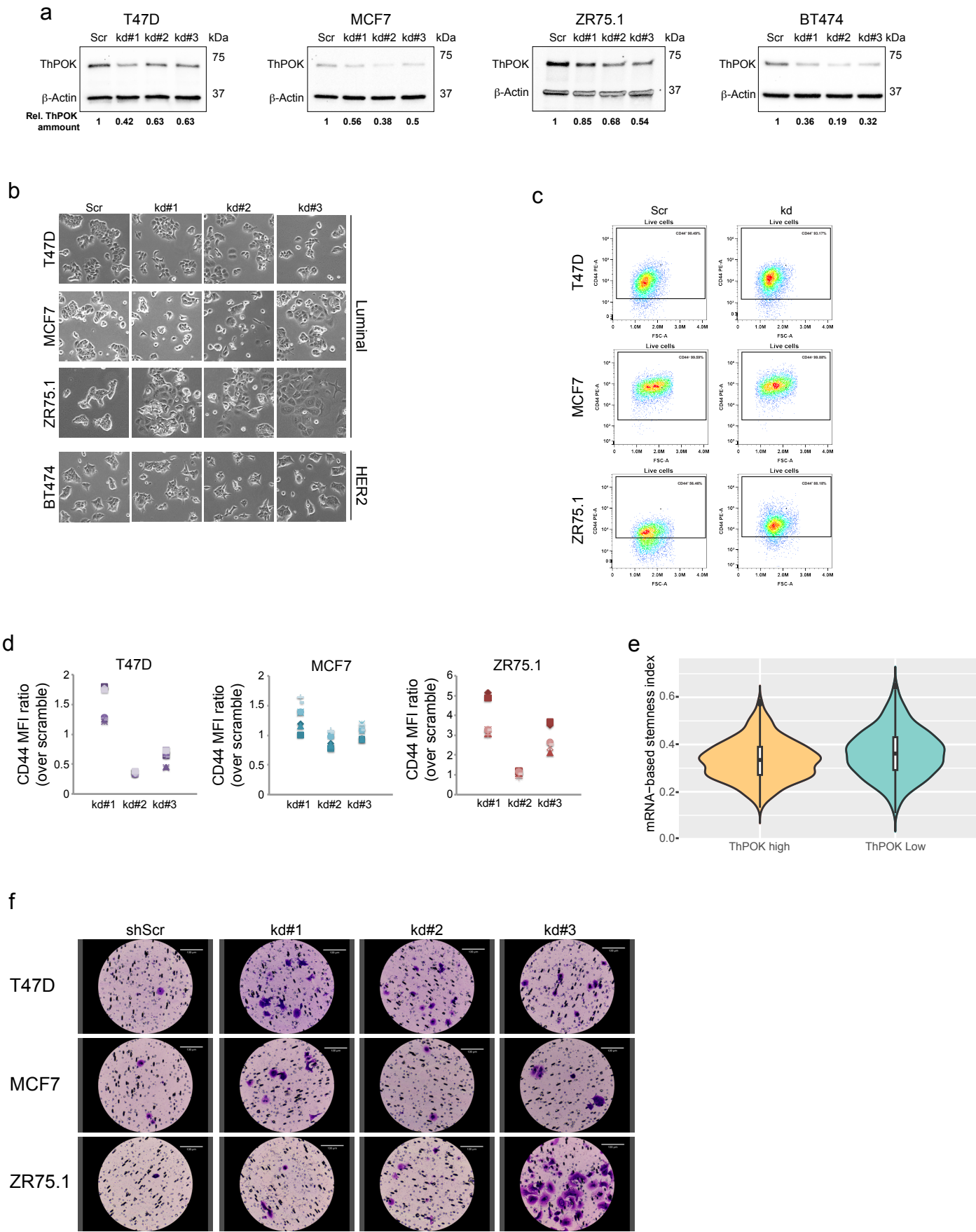

Supplement: Supplementary file 6 — Supplementary file6 (PDF 7620 KB) [file 18_2025_5913_MOESM6_ESM.pdf]

Supplementary Figure 7

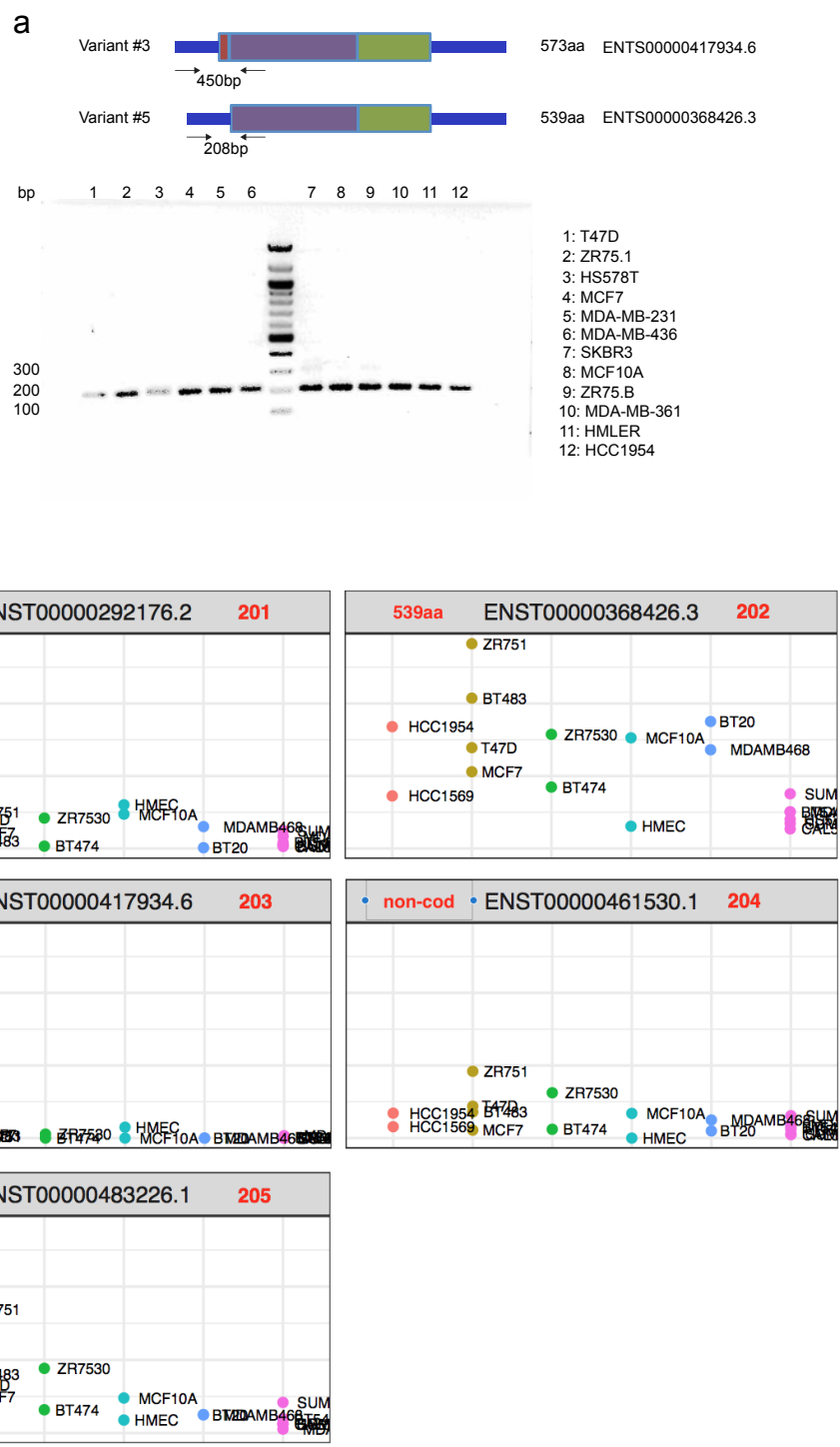

Supplement: Supplementary file 7 — Supplementary file7 (PDF 663 KB) [file 18_2025_5913_MOESM7_ESM.pdf]

Supplementary Figure 8

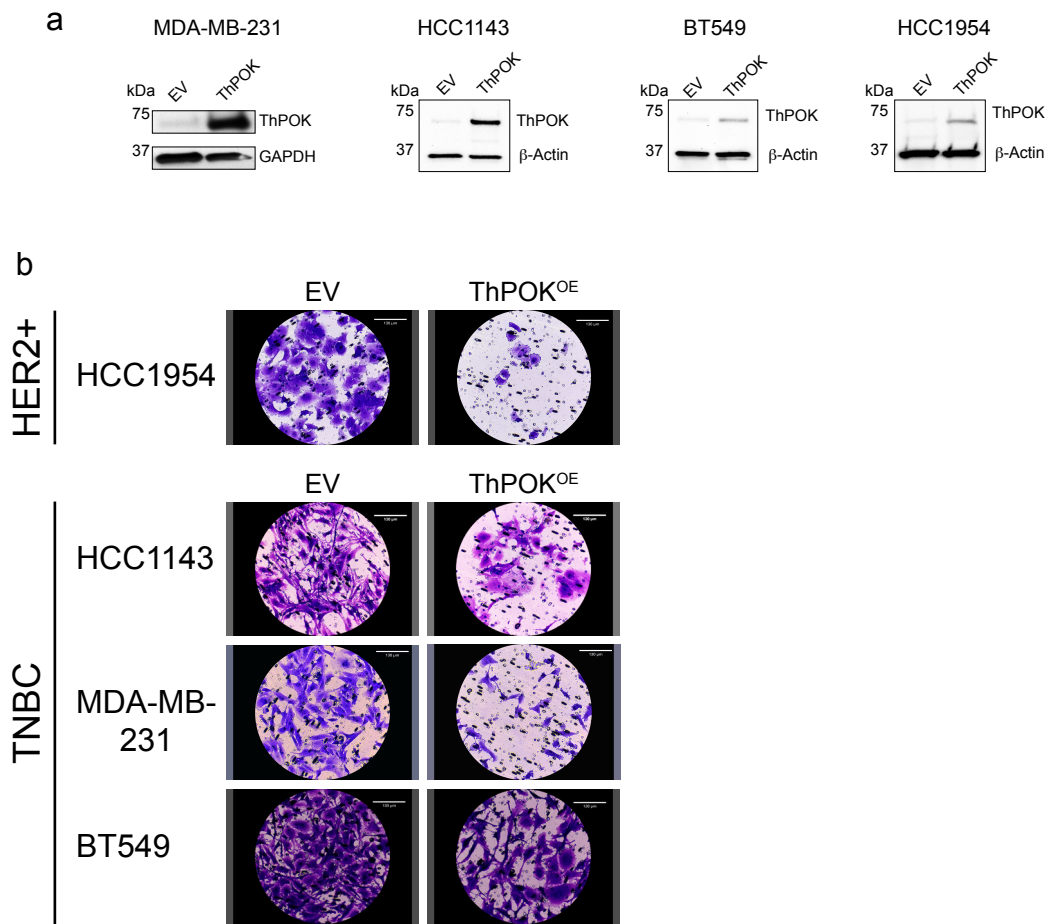

Supplement: Supplementary file 8 — Supplementary file8 (PDF 14154 KB) [file 18_2025_5913_MOESM8_ESM.pdf]

Supplementary Figure 9

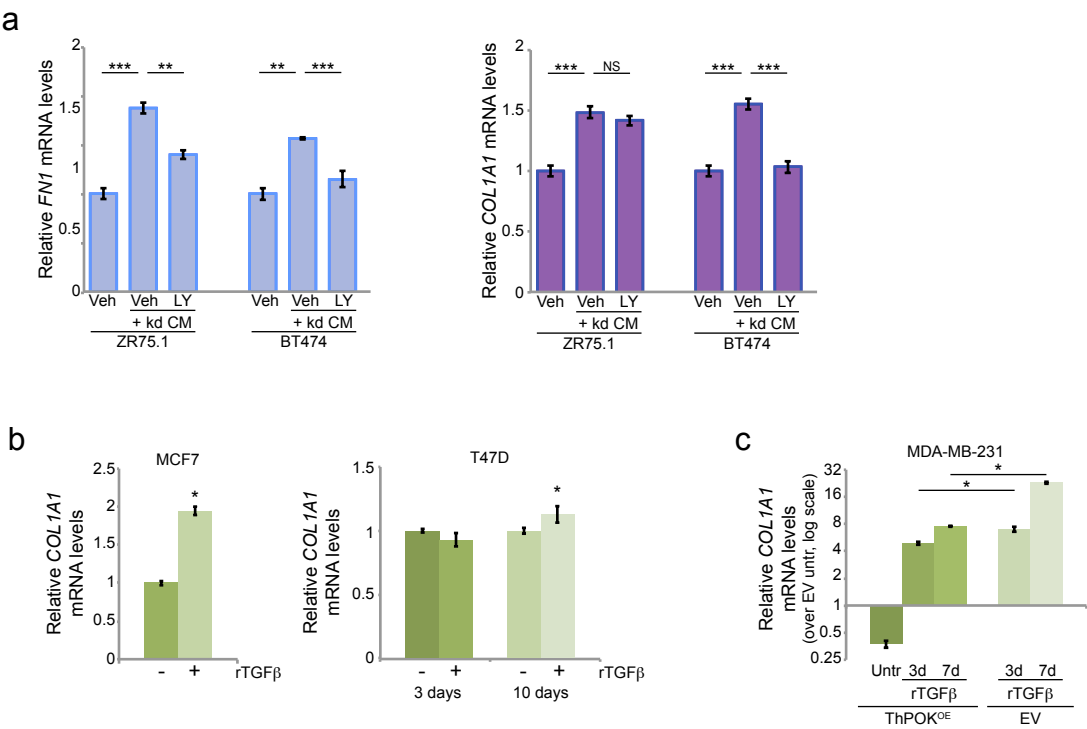

Supplement: Supplementary file 9 — Supplementary file9 (PDF 427 KB) [file 18_2025_5913_MOESM9_ESM.pdf]
