# Supplementary material for: Super-enhancer profiling reveals ThPOK/ZBTB7B, a CD4+ cell lineage commitment factor, as a master regulator that restricts breast cancer cells to a luminal non-migratory phenotype
Source: Cell Mol Life Sci. 2025 Nov 13;82(1):397. doi: 10.1007/s00018-025-05913-4 (PMC12615902; doi:10.1007/s00018-025-05913-4)

Supplementary Figure 2

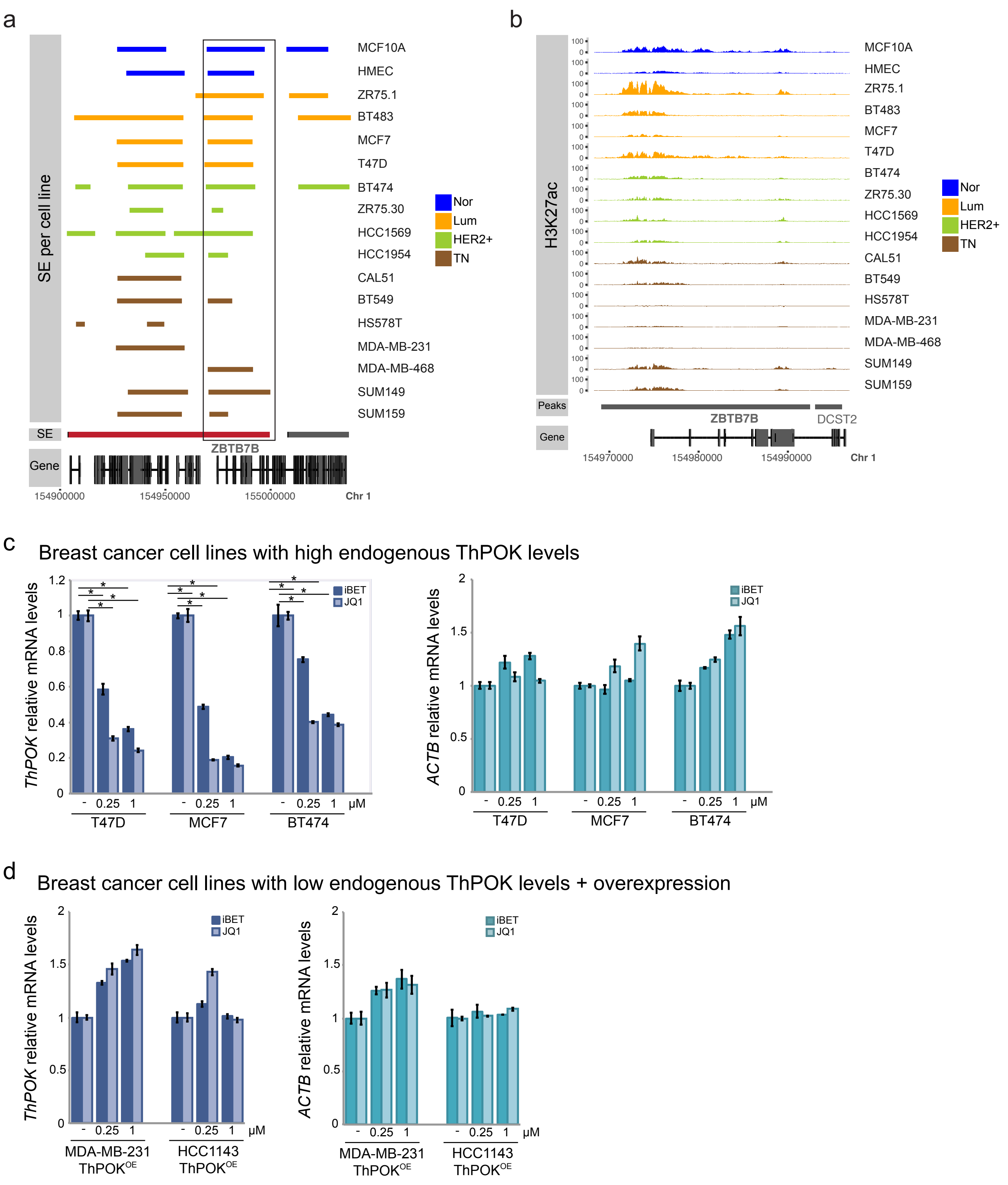

Supplement: Supplementary file 2 — Supplementary file2 (PDF 1040 KB) [file 18_2025_5913_MOESM2_ESM.pdf]
